# Functional Inactivation of the Genome-Wide Association Study Obesity Gene Neuronal Growth Regulator 1 in Mice Causes a Body Mass Phenotype

**DOI:** 10.1371/journal.pone.0041537

**Published:** 2012-07-23

**Authors:** Angela W. S. Lee, Heidi Hengstler, Kathrin Schwald, Mauricio Berriel-Diaz, Desirée Loreth, Matthias Kirsch, Oliver Kretz, Carola A. Haas, Martin Hrabě de Angelis, Stephan Herzig, Thomas Brümmendorf, Martin Klingenspor, Fritz G. Rathjen, Jan Rozman, George Nicholson, Roger D. Cox, Michael K. E. Schäfer

**Affiliations:** 1 MRC Mammalian Genetics Unit, Harwell, Oxfordshire, United Kingdom; 2 Institute of Anatomy and Cell Biology, Center for Neurosciences, University of Freiburg, Freiburg, Germany; 3 Faculty of Biology, University of Freiburg, Freiburg, Germany; 4 Joint Division Molecular Metabolic Control, German Cancer Research Center (DKFZ), Center for Molecular Biology (ZMBH) and University Hospital, Heidelberg, Germany; 5 Experimental Epilepsy Group, Neurocenter, University Clinic Freiburg, Freiburg, Germany; 6 German Mouse Clinic, Helmholtz Zentrum München, Neuherberg, Germany; 7 Center of Life and Food Sciences Weihenstephan, Technische Universität München, Freising, Germany; 8 Max-Delbruck-Centre for Molecular Medicine, Berlin, Germany; 9 Molecular Nutritional Medicine, Else Kröner-Fresenius Center and the Zentralinstitut für Ernährungs- und Lebensmittelforschung (ZIEL) Research Center for Nutrition and Food Sciences, Technische Universität München, Freising, Germany; 10 Department for Anesthesiology, University Medical Center and Focus Program Translational Neuroscience (FTN), Johannes Gutenberg-University Mainz, Mainz, Germany; 11 Department of Statistics, University of Oxford, Oxford, United Kingdom; Montreal Diabetes Research Center, Canada

## Abstract

To date, genome-wide association studies (GWAS) have identified at least 32 novel loci for obesity and body mass-related traits. However, the causal genetic variant and molecular mechanisms of specific susceptibility genes in relation to obesity are yet to be fully confirmed and characterised. Here, we examined whether the candidate gene *NEGR1* encoding the neuronal growth regulator 1, also termed neurotractin or Kilon, accounts for the obesity association. To characterise the function of NEGR1 for body weight control *in vivo*, we generated two novel mutant mouse lines, including a constitutive NEGR1-deficient mouse line as well as an ENU-mutagenised line carrying a loss-of-function mutation (*Negr1*-I87N) and performed metabolic phenotypic analyses. Ablation of NEGR1 results in a small but steady reduction of body mass in both mutant lines, accompanied with a small reduction in body length in the *Negr1*-I87N mutants. Magnetic resonance scanning reveals that the reduction of body mass in *Negr1*-I87N mice is due to a reduced proportion of lean mass. *Negr1*-I87N mutants display reduced food intake and physical activity while normalised energy expenditure remains unchanged. Expression analyses confirmed the brain-specific distribution of NEGR1 including strong expression in the hypothalamus. *In vitro* assays show that NEGR1 promotes cell-cell adhesion and neurite growth of hypothalamic neurons. Our results indicate a role of NEGR1 in the control of body weight and food intake. This study provides evidence that supports the link of the GWAS candidate gene *NEGR1* with body weight control.

## Introduction

Neuronal growth regulator 1 (*NEGR1*), also termed neurotractin [1] or Kilon [2], is among the genes in the expanding list of common obesity loci recently identified in three independent human genome-wide association studies (GWAS) studies [3], [4], [5]. The three associated single nucleotide polymorphisms (SNPs; rs3101336, rs2568958 and rs2815752) lie approximately 60 kb upstream of *NEGR1*, flanking two regions of deletion polymorphisms that segregate on distinct haplotypes. These deletions remove conserved elements upstream of *NEGR1* and are associated with increased body mass index (BMI) [5]. Since the GWAS discovery of this novel obesity locus, the association of the variants within *NEGR1* loci has been replicated in various genotyping studies for body mass, BMI and other obesity-related traits such as birth weight, subcutaneous fat mass and infancy weight gain [6], [7], [8], [9], [10], [11]. However, other studies have failed to replicate the association in other specific populations [12], [13], [14].

So far, many of the GWAS hits have pointed to a role of the central nervous system (CNS) in obesity and have identified proven functional obesity genes such as *MC4R*, *SH2B1* and *BDNF*. Disruption of *Mc4r*, *Sh2b1* and *Bdnf* in mice all result in hyperphagia and/or obesity [5]. However, the function of other neural-specific candidate genes such as *NEGR1*, *TMEM18* and *KCTD15* are yet to be proven *in vivo*.

NEGR1 is a cell adhesion molecule of the immunoglobulin (Ig) superfamily that belongs to the IgLON subgroup. In mammals, this subgroup consists of NEGR1/neurotractin/Kilon (kindred of IgLON), Neurotrimin (NTM), OPCML/OBCAM (opioid-binding protein/cell adhesion molecule) and LSAMP/LAMP (limbic system associated membrane protein). IgLON members share common features consisting of three Ig-like C2-type domains and a glycosylphosphatidylinositol (GPI)-anchor attachment signal. Several splice variants have been identified which differ in their signal peptides [15], [16], number of Ig-like domains [1], or become secreted due to alternative splicing of the region encoding for GPI-mediated anchorage [17]. IgLONs localise to distinct but overlapping populations of neurons. Dependent on the cellular context, IgLONs have been proposed to either enhance or inhibit neurite growth and synapse formation, respectively [1], [18], [19], [20], [21], [22]. Phenotypic analyses of mice deficient for the IgLON member LSAMP revealed abnormalities in social behaviour, impaired synaptic plasticity and spatial memory [23], [24], [25]. The underlying mechanisms are unclear but may relate to alterations in neuronal connectivity, mineralocorticoid receptor expression and GABA(A) receptor subunit expression [23], [24], [25].

Although predominantly expressed in neurons, experimental brain lesion in rodents induces expression of NEGR1/neurotractin/Kilon [22] and the related IgLON member OBCAM [26] in reactive astrocytes. In addition, IgLONs have been linked to different types of cancer and may act as tumor suppressor genes [27], [28], [29], [30], [31]

To date, the *in vivo* function of NEGR1 is unknown and functional models that confirm the proposed role of NEGR1 in the development of obesity have not been reported. We describe here two mouse alleles of *Negr1*, both of which ablate NEGR1 function and result in a reduction of body mass. Phenotypic analysis of the ENU mutant, *Negr1*-I87N, has revealed the weight loss to be attributed to a reduction in lean body mass and a small reduction in body length. Further analysis of these novel mouse models will enable us to study the contribution of NEGR1 to body weight control in mammals at a molecular level.

## Results

### Generation of two mouse *Negr1* alleles

**Figure 1:**
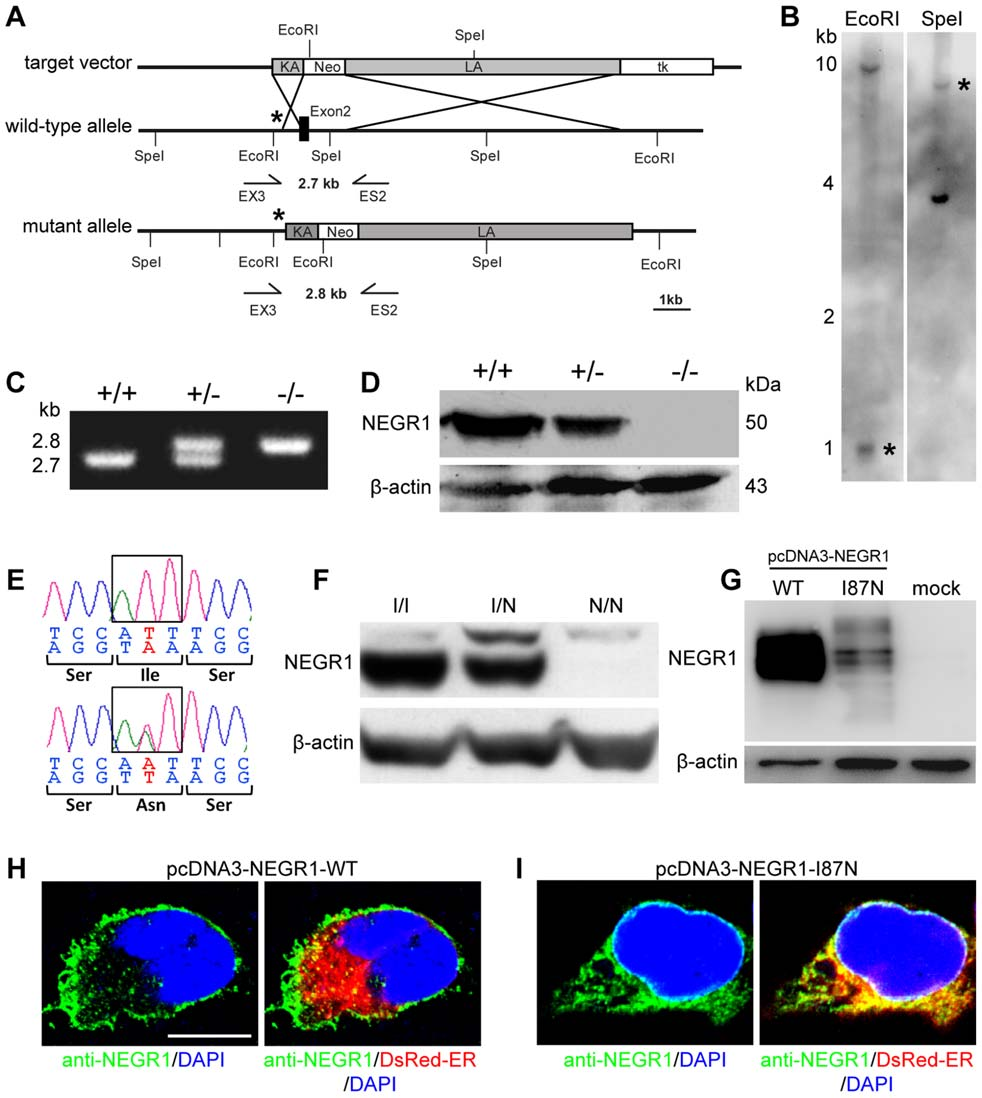
Ablation of NEGR1 in mouse. **A.** Generation of *Negr1* knockout mice. Schematic diagram of the targeting vector, the wild-type and mutant alleles. Only relevant restriction sites are shown. The positions of the external southern blot probe, as well as the PCR primers, are indicated by asterisks and arrows, respectively. **B.** Genomic southern blot using the restriction enzymes EcoRI and SpeI. Bands resulting from introduction of a new EcoRI site and deletion of a SpeI site are indicated by asterisks. **C.** PCR genotyping of transgenic mice wild-type (+/+), heterozygous (+/−) and homozygous (−/−) mice. **D.** Western blot probed with antibodies specific to NEGR1 and β-actin showing complete loss of NEGR1 protein in knockout (−/−) mice. **E.** Sequencing of genomic DNA reveals a T-A mutation that converts an isoleucine residue to asparagine at position 87 (I87N). **F.** Western blots of brain lysate from *Negr1*-I87N mice probed with an anti-NEGR1 antibody. **G.** NEGR1-immunoblotting of NSC-34 cell lysates overexpressing NEGR1-WT, NEGR1-I87N, and mock-transfected cells. **H–I.** The *Negr1*-I87N mutation causes ER retention of NEGR1. Confocal images showing NSC-34 cells co-expressing NEGR1-WT (H) or NEGR1-I87N (I) together with DsRed-ER. NEGR1-WT is predominantly localized at the plasma membrane whereas distribution of NEGR1-I87N clearly overlaps with the DsRed-fluorophore-labeled ER. Nuclei are visualised by DAPI. Scale: 10 µm.

To test the effect of inactivating *Negr1 in vivo*, we generated two alleles that ablate NEGR1 expression in mice. The first allele is a constitutive knockout (KO), in which the mouse *Negr1* gene was mutated in embryonic stem (ES) cells by replacement of exon 2, which encodes the first Ig-like domain and the 3′ exon/intron splice site, with a neomycin resistance cassette (Fig. 1A). Three ES cell clones, verified by southern blotting (Fig. 1B) and genomic PCR (Fig. 1C) were used to generate chimeric mice which were crossed to C57BL/6J mice for germline transmission of the KO allele. All three clones were transmitted into the germline. Western blot analysis using antibodies specific to NEGR1 demonstrate the reduction of NEGR1 in heterozygous mutants and absence of the protein in homozygous mutants (Fig. 1D).

The second allele was identified by screening for ENU-induced mutations in *Negr1* using the Harwell ENU DNA archive [32]. We re-derived a mutant that carries a point mutation (T260A) resulting in a non-synonymous substitution from isoleucine (I) to asparagine (N) (Fig. 1E). The I87N residue lies within the first Ig-like C2-type domain in close proximity to the signal peptide (Fig. S1A). NEGR1 shares 95% homology between mouse and human. The I87 residue is conserved across a wide range of mammalian species (Fig. S1B), as well as lower vertebrates such as *Gallus gallus* (chicken), *Xenopus laevis* (frog) and *Tetraodon nigroviridis* (pufferfish). Both mouse lines are viable and fertile with intercross breeding yielding the expected Mendelian ratio (data not shown).

### The *Negr1*-I87N mutation affects protein expression and leads to ER retention of NEGR1

NEGR1 protein has a molecular mass of 37 kDa but contains several N-linked glycosylation sites which results in a molecular mass of approximately 50 kDa [22]. The I87N mutation appears to completely abolish *in vivo* expression of fully glycosylated NEGR1 as confirmed by immunoblots (Fig. 1F). Similarly, when overexpressed in the Neuroblastoma×spinal cord hybrid cell line NSC-34 [33], which does not express detectable amounts of endogenous NEGR1 protein, immunoblot analyses revealed substantial levels of NEGR1-WT but remarkably low levels of NEGR1-I87N protein (Fig. 1G).

Missense mutations of conserved amino acids may cause protein misfolding and retention in the endoplasmic reticulum (ER) thereby disrupting the function of cell adhesion molecules [34], [35]. To address this issue we compared the subcellular localization of wild-type and NEGR1-I87N in NSC-34 cells. Using this overexpression approach, we observed that wild-type NEGR1 is predominantly localized at the plasma membrane (Fig. 1H) whereas NEGR1-I87N overlaps with the ER marker DsRed-ER (Fig. 1I). These results indicate that the *Negr1*-I87N mutation interferes with protein expression, cell surface trafficking and thus function of NEGR1.

### Loss of NEGR1 function causes a reduction in body mass

**Figure 2:**
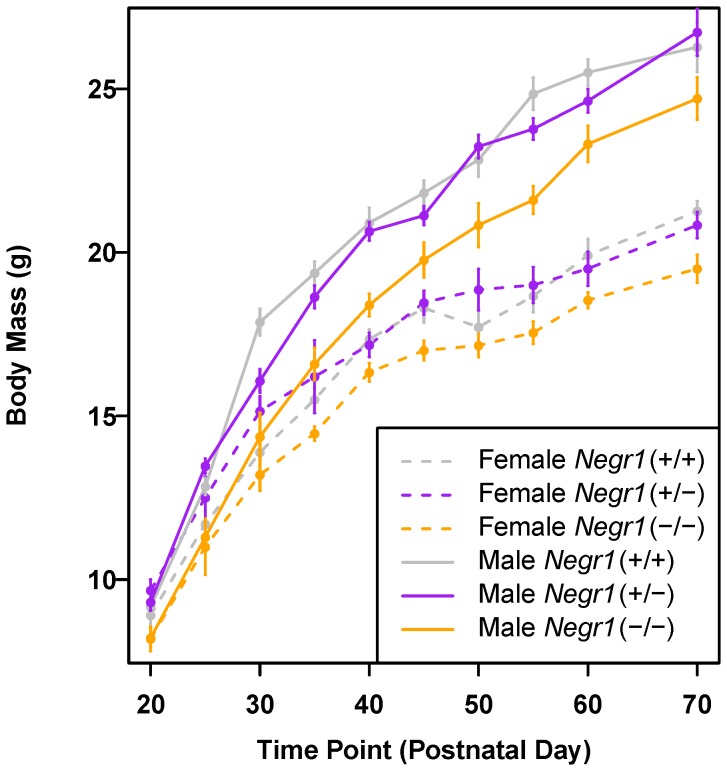
Mice deficient of NEGR1 exhibit reduced body mass and lean mass. Growth curve of *Negr1*-KO females (*Negr1^+/+^*, *n*∼13; *Negr1^+/−^*, *n*∼7; *Negr1^−/−^*, *n*∼10), and males (*Negr1^+/+^*, *n*∼17; *Negr1^+/−^*, *n*∼21; *Negr1^−/−^*, *n*∼15) on standard chow diet measured across 10 weeks. Data shown are mean +/− SEM within each sex-genotype group at each time point.

According to findings from GWAS, the three SNP variants in the *NEGR1* locus are associated with body mass and BMI in human with a moderate effect size. To determine if the loss of NEGR1 has an effect on body mass, the *Negr1*-KO and *Negr1*-I87N mice were weighed over time for 10 and 18 weeks, respectively. The KO allele confers an overall reduction in body mass (Fig. 2 and Table 1, repeated measures ANOVA, males and females *P*<0.0001). The differences become apparent after weaning, around 3–4 weeks of age (Fig. 2), suggesting that there is not a defect in suckling or feeding during the postnatal period. *Negr1*-KO mutant mice fed a standard chow diet, displayed up to 8% and 13% reduction in total body mass in females and males, respectively (Fig. 2). Genotypic effects were estimated and tested within individual time points (time-by-time ANOVA models [36]; see Materials and Methods). In models fitted collectively to data from males and females, the number of time points with significantly reduced body weight (Bonferroni corrected *P*<0.05) was 8 out of 10 for *Negr1*-KO, indicating a clear genotypic effect on body mass (Table S1).

**Table 1:** Repeated measures ANOVA—results for *Negr1*-KO experiment.

		Heterozygote - WT^i^		Heterozygote - WT^ii^		
Phenotype	Model	Estimate	S.E.iii	Estimate	S.E.	p-value^iv^
Body mass	Males	−0.492	0.273	−2.174	0.291	1.09E−11
Body mass	Females	0.269	0.305	−1.364	0.285	6.66E−07
Body mass	Males and Females	−0.219	0.209	−1.854	0.213	8.60E−17

i ‘Heterozygote – WT’ denotes the mean difference between the heterozygote and WT genotypic classes (i.e. 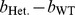 in the notation developed in Materials and Methods).

ii ‘Homozygote – WT’ denotes the mean difference between the heterozygote and WT genotypic classes (i.e. 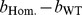 in the notation developed in Materials and Methods).

iii Standard error.

iv Nominal p-values for the test of the null hypothesis of no genotypic effect (described in Materials and Methods).

### Reduced body mass phenotype in *Negr1*-I87N mice manifests upon high-fat diet

**Figure 3:**
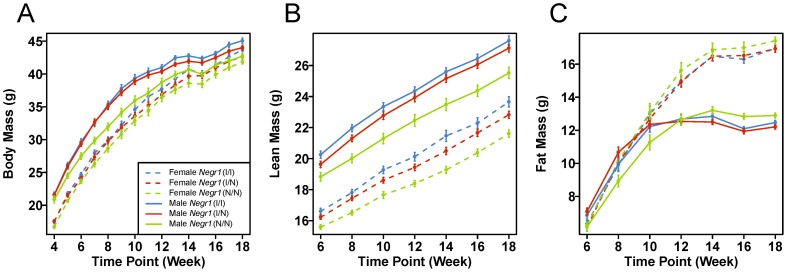
*Negr1*-I87N mutants display altered body mass and composition. **A–D**. Body mass (A), Lean mass (B), fat mass (C) of wild type (female, n = 28; male, n = 22), heterozygous (female, n = 52; male, n = 51) and homozygous (female, n = 28; male, n = 24) *Negr1*-I87N mice fed on high-fat diet measured across 18 weeks. Data shown are mean +/− SEM within each sex-genotype group at each time point.

For the *Negr1*-I87N allele, initially a cohort was split into standard chow and high-fat diet groups. In this generation (after 2 consecutive backcrosses to C3H/HeH), the reduction in body mass in *Negr1*-I87N mutants becomes greater when mice were fed with high-fat diet (45% kcal fat) from 6-wk (data not shown). To further study this, a new cohort (after 6 consecutive backcrosses to C3H/HeH) was bred and fed a high-fat diet. All the phenotypic data on *Negr1*-I87N mice presented hereafter are based on data collected from mice fed with a high-fed diet. Homozygous *Negr1*-I87N mice display a reduction of total body mass of approximately 4.5% (up to 5.8%) and 6.5% (up to 9.6%) compared to wild-type in females and males, respectively (Fig. 3A). Repeated measures ANOVA confirmed a statistically significant reduction in body mass for the *Negr1*-I87N allele in males (*P* = 0.0005) with a trend in females (*P* = 0.082) and increased significance (*P* = 0.0002) when males and females were combined (Table 2).

**Table 2:** Repeated measures ANOVA—results for *Negr1*-I87N experiment.

		Heterozygote - WT^v^		Homozygote - WT^vi^		
Phenotype	Model	Estimate	S.E.vii	Estimate	S.E.	p-valueviii
Body mass	Males	−0.531	0.57	−2.445	0.66	0.000507
Body mass	Females	−0.638	0.58	−1.507	0.67	0.0819
Body mass	Males and Females	−0.58	0.41	−1.936	0.474	0.000165
Lean Mass	Males	−0.514	0.385	−1.94	0.446	5.88E−05
Lean Mass	Females	−0.649	0.298	−1.687	0.344	1.46E−05
Lean Mass	Males and Females	−0.585	0.24	−1.804	0.277	1.18E−09
Fat Mass	Males	0.026	0.236	−0.169	0.274	0.69
Fat Mass	Females	−0.027	0.349	0.347	0.403	0.541
Fat Mass	Males and Females	0.002	0.219	0.111	0.253	0.865
% Fat Mass	Males	0.57	0.468	1.302	0.541	0.0578
% Fat Mass	Females	0.474	0.55	2.385	0.636	0.00043
% Fat Mass	Males and Females	0.528	0.37	1.89	0.427	3.30E−05

v ‘Heterozygote – WT’ denotes the mean difference between the heterozygote and WT genotypic classes (i.e. 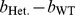 in the notation developed in Materials and Methods).

vi ‘Homozygote – WT’ denotes the mean difference between the heterozygote and WT genotypic classes (i.e. 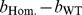 in the notation developed in Materials and Methods).

vii Standard error.

viii Nominal p-values for the test of the null hypothesis of no genotypic effect (described in Materials and Methods).

In time-by-time ANOVA models (see above) the number of time points with significantly reduced body weight (Bonferroni corrected *P*<0.05) was 8 out of 15 for *Negr1*-I87N, again indicating a clear genotypic effect on body mass (Table S1). The difference between homozygotes and wild-type littermate males increased up to 9 weeks and then reduced again suggesting different rates of linear growth (Table S1 and Fig. 3A).

### The *Negr1*-I87N mutation affects body mass composition

We next examined changes in body composition that may underlie the body mass reduction in the high-fat diet group. Fat mass and lean mass were measured every 2 weeks by whole-body scanning quantitative magnetic resonance. Mice homozygous for the *Negr1*-I87N allele showed a significant reduction of approximately 8% in lean mass in both females and males (Fig. 3B). In a repeated measures ANOVA analysis this was highly significant (*P*<0.0001) for a genotype effect on lean mass in grams in males and females (Fig. 3B and Table 2). Comparing individual time points and correcting for multiple testing this was significant at all except the first time point in males (Table S1). Total gram fat mass was not affected in *Negr1*-I87N mice (Fig. 3C, Table 2 and Table S1).

**Figure 4:**
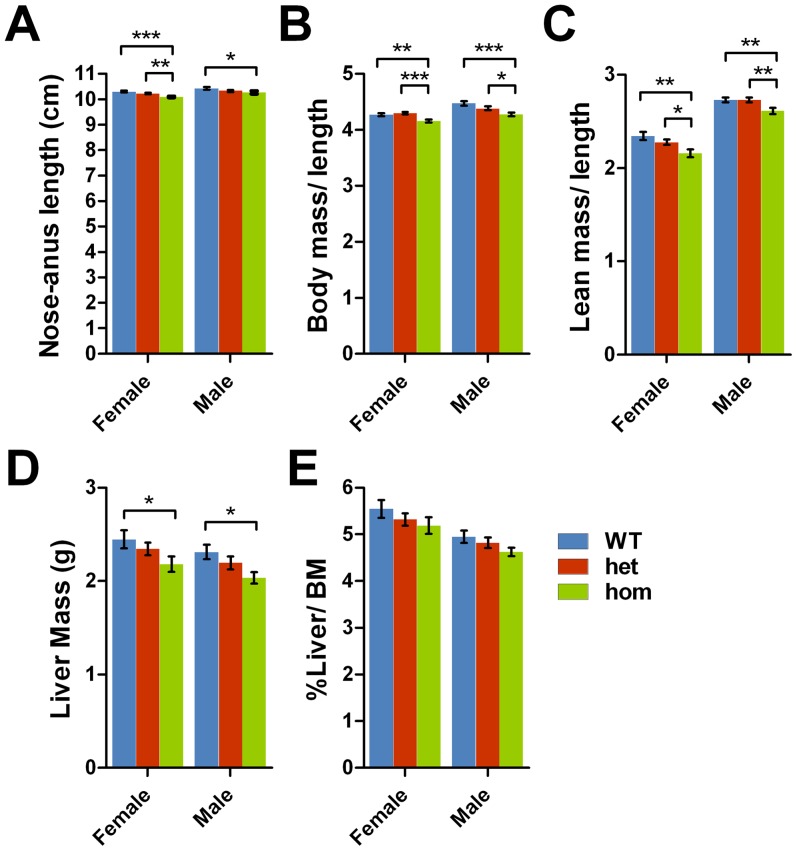
*Negr1*-I87N mutants have altered body mass composition. **A–G**. Body length (A), body mass (B) and lean mass (C) normalised to body length, liver mass (D) and percentage of liver mass to total body mass (E) of wild-type (female, n = 23; male, n = 16), heterozygous (female, n = 37; male, n = 36) and homozygous (female, n = 17; male, n = 19) *Negr1*-I87N mice measured at 22 weeks in females and males. All data are presented as mean ± SEM. Student's *t*-test was carried out between groups, *, *P*<0.05; **, *P*<0.01; ***, *P*<0.001.

To test whether the mutants are smaller in body size, the nose-anus length was measured in anaesthetized animals at 22-wk. Although there is a statistically significant reduction in body length in *Negr1*-I87N homozygotes, the reduction is only 2% and 1.5% in female and male, respectively (Fig. 4A). When body mass and lean mass are normalised to nose-anus length, the reduction in homozygous mice remains significant (Fig. 4B,C), suggesting that the changes in body mass and lean mass cannot be entirely explained by a reduction in body length.

**Figure 5:**
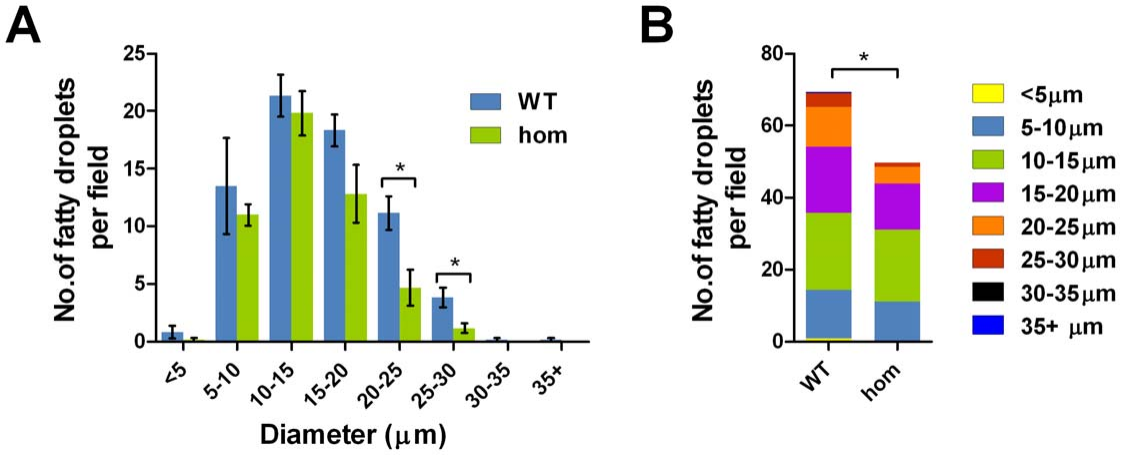
Ablation of NEGR1 causes reduction in fatty droplets. **A**,**B**. Number of triglyceride droplets classified into ranges of size in diameter (µm) in hepatocytes of female *Negr1*-I87N mice (n = 3 mice per genotype). All data are presented as mean ± SEM. Student's *t*-test (2-tailed) was carried out between groups, *, *P*<0.05; **, *P*<0.01; ***, *P*<0.001.

In addition to the reduction in lean mass, whole liver from *Negr1*-I87N mutants are lighter comparing to wild-type at 22-wk in both sexes, although, taking account of body weight reduction reduces this to a trend (Fig. 4D,E). As a result of chronic high-fat diet, both wild-type and mutant animals display fatty liver with appearance of triglyceride droplets within hepatocytes. In *Negr1*-I87N homozygotes, however, the number of triglyceride droplets is significantly reduced (Fig. 5 A and B), which may partly explain the reduction in liver mass in comparison to wild-type.

### *Negr1*-I87N mice display reduced physical activity and food intake but unchanged normalised energy expenditure

**Figure 6:**
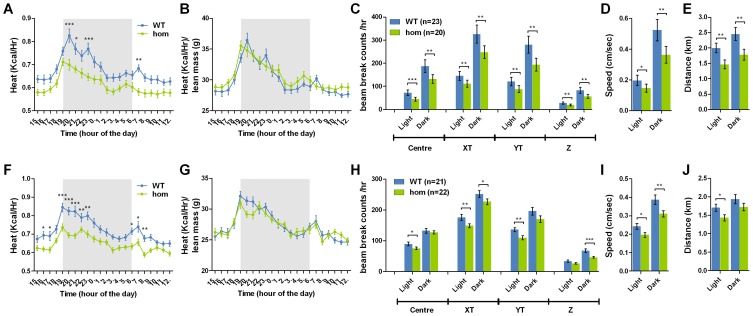
*Negr1*–I87N mice have unchanged energy expenditure relative to lean mass and reduced physical activity. **A,B,F,G**. Energy expenditure (A, F) normalised to lean mass (B, G) over a 22-hr period during light and dark phases in 16-week old wild-type (female, n = 23; male, n = 21) and homozygotes (female, n = 20; male, n = 22) females (A–B) and males (F–G). **C–E, H–J**. Physical activity as measured by the average number of beambreak counts in various different dimensions (C, H), average speed (D, I) and total distance travelled (E, J) in 16-week old wild-type (female, n = 23; male, n = 21) and homozygotes (female, n = 20; male, n = 22) females (C–E) and males (H–J). All data are presented as mean ± SEM. Two-tailed Student's *t*-test was carried out between groups, *, *P*<0.05; **, *P*<0.01; ***, *P*<0.001. Bonferroni correction was applied to adjust for multiple measurements (A,B,F,G).

Previous studies have shown that changes in energy expenditure are often explained by changes in underlying lean mass [37], [38], [39]. We used indirect calorimetry to assess metabolic rate in 12- and 16-wk-old mice. At both time points, we observed a marked reduction in energy expenditure (heat, kcal h^−1^) during both light and dark periods in *Negr1*-I87N homozygotes (Fig. 6A,F). This difference was abolished after normalisation to lean mass (Fig. 6B,G). Consistently, no significance is found when energy expenditure is correlated against body mass and lean mass by linear regression analysis in both light and dark periods (Fig. S2A–H). Data points of wild-type and homozygous mice follow almost a linear relationship between lean mass and heat, indicating that the reduction in energy expenditure is explained by the reduction in lean mass (Fig. S2).

Interestingly, there is also an overall reduction in physical activity as measured by the number of breaks in infrared beams (Fig. 6C,H), average speed of movements (Fig. 6D,I) and total distance travelled (Fig. 6E,J) within 24 hrs in a photobeam-based activity monitoring system, consistent with the reduced energy expenditure before normalisation (Fig. 6A,F).

**Figure 7:**
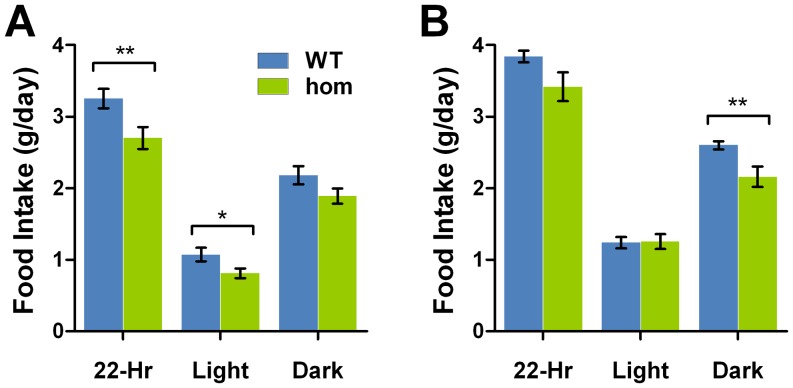
Ablation of NEGR1 causes reduction in food intake. **A,B.** Food intake over 24 hrs measured at 16-weeks in *Negr1*-I87N female (A) (WT, n = 23; hom, n = 20) and male (B) (WT, n = 21; hom, n = 22). All data are presented as mean ± SEM. Student's *t*-test (2-tailed) was carried out between groups, *, *P*<0.05; **, *P*<0.01; ***, *P*<0.001.

We next tested several parameters related to feeding behaviour in *Negr1*-I87N mice. Mice homozygous for the *Negr1*-I87N allele exhibit a reduction in food consumption in comparison to wild-type measured over 24 hrs in both females (Fig. 7A) and males (Fig. 7B). Interestingly, the daily energy loss via faeces was significantly reduced in homozygous male mice as measured by bomb calorimetry, despite a lack of difference in dried faeces mass (–C).

### Dependence of body mass on genotype and food intake

The mutual dependence between body mass, sex, genotype, food intake, energy expenditure, and physical activity was investigated by fitting a mixed graphical model [40], [41]. Such a model characterizes the conditional dependence structure amongst the variables, and has the important property that it can handle quantitative and qualitative variables (see Materials and Methods). Under the best fitting model, represented by the graph in Figure S4, body mass was conditionally independent of sex, energy expenditure, and physical activity, given food intake and genotype (so, for example, under the model, food intake explains the association between sex and body mass).

The relationship between body mass, genotype, and food intake was further explored by fitting an ordinary linear model, in which body mass was the response variable, and food intake and genotype were the explanatory variables (details of the model fit are in Table S2; see also Materials and Methods). Food intake and genotype collectively explained 36% of variation in body mass (i.e. multiple 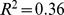). By decomposing 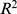, it was found that 22% of variation in body mass was explained by food intake but not genotype, 7% was explained by genotype but not food intake, and the remaining 7% of 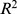 was explained by variation shared by genotype and food intake (Materials and Methods).

### NEGR1 expression in the hypothalamus

**Figure 8:**
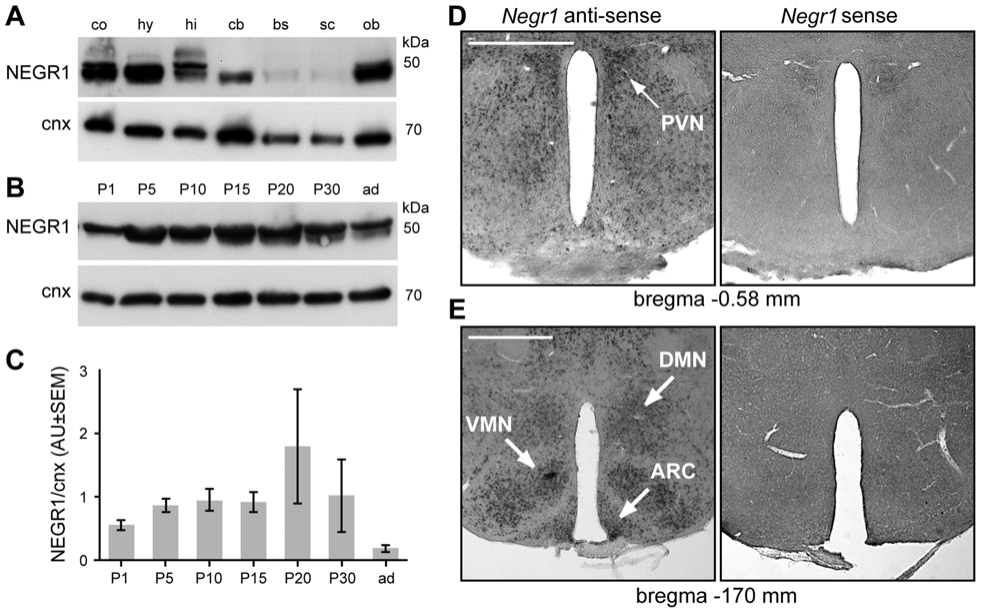
Expression of NEGR1. **A.** NEGR1 protein expression in different CNS regions from adult mice. **B.** Developmental expression regulation of NEGR1 in the postnatal hypothalamus. Blots were probed with antibodies specific to NEGR1 and ubiquitously expressed calnexin (cnx). **C.** Semiquantitative assessment of hypothalamic NEGR1 protein expression relative to cnx during postnatal development. **D–E.** Distribution of *Negr1* mRNA in the adult murine hypothalamus in reference to bregma (n = 4 mice/condition). Scale bars: 500 µm (D–E); ad: adult; co: cerebral cortex; hy: hypothalamus; hi: hippocampus; cb: cerebellum; bs: brainstem; sc: spinal cord; ob: olfactory bulb; cnx: calnexin; PVN: paraventricular nucleus; DMN: dorsomedial nucleus; VMN: ventromedial nucleus; ARC: arcuate nucleus.

GWAS have suggested a potential role of *NEGR1* in the central nervous control of body weight [3], [5] because of its predominant expression in the rodent CNS [22], [42]. Our observation of reduced food intake in mutant mice also suggests that food control centres in the brain may be involved. Although *NEGR1* has been also reported to be expressed at the mRNA level in various peripheral tissues [43], we were unable to detect substantial amounts of NEGR1 protein in peripheral tissues by immunoblotting (Fig. S5A). To characterise the expression of NEGR1 protein in the murine CNS we performed immunoblot analyses of different CNS regions (Fig. 8A). In agreement with previous studies [22], [42], we found NEGR1 strongly expressed in various brain regions including the cerebral cortex, hippocampus and the olfactory bulb. Moreover, we observed strong expression of NEGR1 in the hypothalamus (Fig. 8A). During postnatal development, hypothalamic NEGR1 expression increased from postnatal stage P1 to P5, was maintained from P5 to P30 but declined in the adult CNS relative to ubiquitously expressed calnexin (Fig. 8B,C). To reveal the spatial distribution of *Negr1* mRNA in the adult hypothalamus, brain sections were processed for *in situ* hybridisation using an anti-sense probe specific for *Negr1* (Fig. 8D,E). Expression of *Negr1* mRNA was found in all hypothalamic nuclei including the paraventricular nucleus (PVN), dorsomedial nucleus (DMN), ventromedial nucleus (VMN) and the arcuate nucleus (ARC) (Fig. 8D,E).

The PVN and the ARC, which can be more clearly distinguished in histological sections than other hypothalamic nuclei, were dissected by laser capture microdissection (LCM) (Fig. S5B,C) and *Negr1* mRNA levels analysed by quantitative PCR (qPCR). These analyses revealed no significant differences for *Negr1* expression between these hypothalamic nuclei (Fig. S5D). Expression regulation for various hypothalamic genes, as those encoding for the anorexigenic neuropeptides NPY and AgrP, is highly dynamic upon fasting [44], [45]. However, in contrast to significantly raised *Npy* mRNA levels, we found no significant regulation of *Negr1* mRNA expression after a fasting period of 24 hrs (Fig. S5D–E).

Together, NEGR1 protein expression is brain-specific and widely distributed. There is remarkably high expression of *Negr1* mRNA in hypothalamic nuclei which is not altered by acute changes in the nutritional state. The developmental expression regulation in the hypothalamus, suggest a role in the nervous system maturation.

### NEGR1 promotes cell-cell adhesion and stimulates neurite growth of hypothalamic neurons

Neural members of the Ig-superfamily have been shown to participate in different aspects of nervous system development including neuronal migration, axon growth and guidance as well as synapse formation and plasticity [46]. Furthermore, GWAS have suggested a potential role of *NEGR1* in the nervous control of body weight [5]. To examine the cellular function of NEGR1 we carried out cell-cell aggregation and neurite growth experiments.

**Figure 9:**
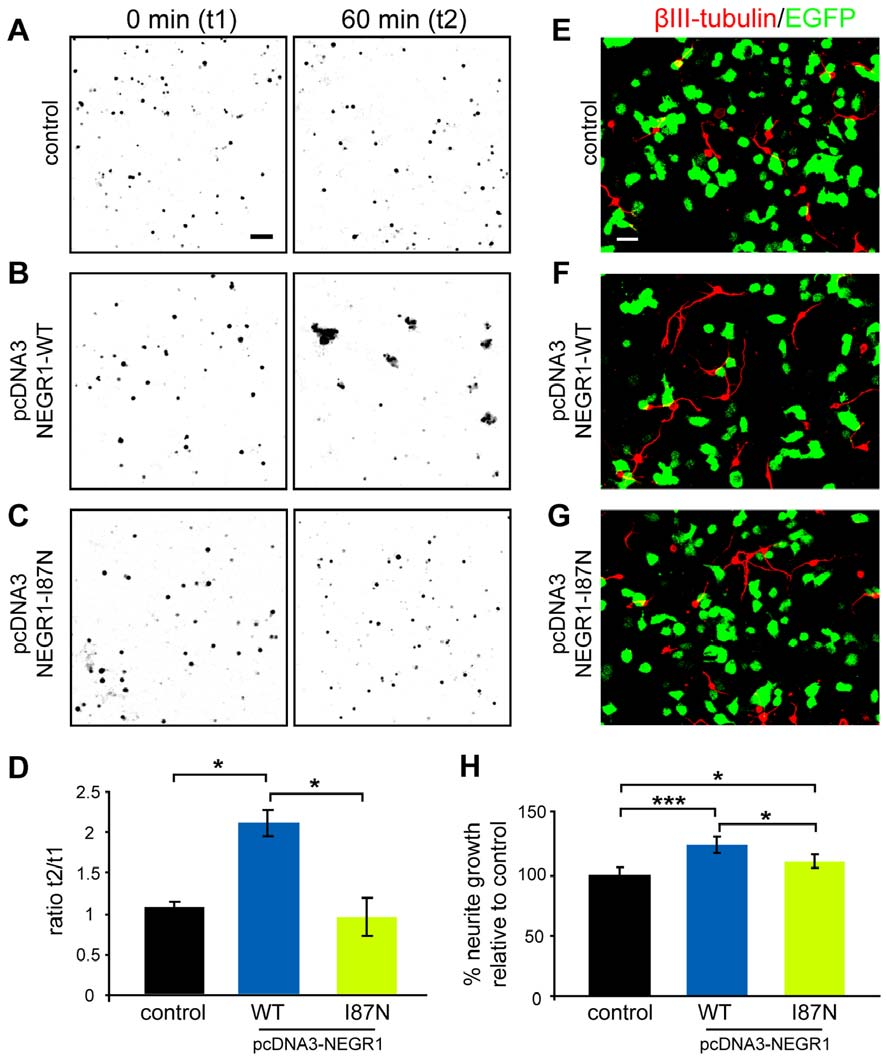
*Negr1*-I87N is a loss-of-function mutation. **A–C.** NSC-34 cells expressing EGFP alone (control) or together with *Negr1*-WT or *Negr1*-I87N mutants at 0 min (t1) and following 60 min (t2) of cell aggregation. **D.** Histogram showing cell aggregation expressed as the ratio of 0 min (t1) and 60 min (t2) time points. EGFP+empty vector (control): 1.06±0.05; NEGR1-WT+EGFP: 2.11±0.2; EGFP+NEGR1-I87N: 0.93±0.2. Data represent means ± sd calculated from three independent experiments. **E–G.** Confocal images showing hypothalamic neurons immunostained for the neuronal marker βIII-tubulin (red) cultured together with transfected NSC-34 cells (green). NSC34 cells were transfected with (E) pEGFP together with an empty pcDNA-vector (control), (F) pEGFP+pcDNA3-NEGR1-WT and (G) EGFP+NEGR1-I87N. **H.** Mean neurite lengths of hypothalamic neurons relative to control (set to 100%). Error bars represent SEM from three independent experiments (∼400 neurites per condition). Two-tailed Student's test; *P<0.05; **P<0.01; *** *P*<0.001. Scale: 100 µm (A), 20 µm (E).

We incubated NSC34 cells co-expressing wild-type or mutant NEGR1 in NSC-34 cells with EGFP as a fluorescent reporter (Fig. 9A–C). Incubation of cells expressing NEGR1-WT/EGFP clearly resulted in the formation of cell aggregates (Fig. 9B) as determined by the area occupied by aggregated cells (Fig. 9D), suggesting that homophilic interaction of NEGR1 promotes cell-cell adhesion *in trans*. As expected, NEGR1-I87N failed to promote formation of aggregates (Fig. 9C and D).

Next, we offered NSC-34 cells expressing NEGR1 as a biological substrate for primary neurons. Given the strong expression of NEGR1 in the hypothalamus we prepared primary hypothalamic neurons from newborn mice. Co-cultures were immunostained after two days of cultivation with antibodies specific to the neuronal marker βIII-tubulin (Fig. 9E–G) and the longest neurites of individual neurons were measured. These analyses revealed that NEGR1-WT stimulates neurite growth of hypothalamic neurons (Fig. 9F), compared to control cells expressing EGFP alone (Fig. 9E) or those expressing NEGR1-I87N (Fig. 9G). However, NEGR1-I87N expressing cells also displayed a weak stimulatory effect on neurite growth, albeit to a clearly lesser extent than NEGR1-WT (Fig. 9G,H). Thus, NEGR1 promotes cell-cell adhesion and stimulates neurite growth of hypothalamic neurons. Together with the data on the *in vivo* expression regulation, these results suggest a role of NEGR1 in neural circuit formation in the hypothalamus.

## Discussion

In this study, we report two novel mouse mutants for studying the GWAS candidate obesity gene *NEGR1*. Importantly, loss of NEGR1 function causes an overall reduction in body mass in our mouse models. The body mass phenotype of both *Negr1*-KO and *Negr1*-I87N mutants develops after weaning, suggesting that there is not a defect in suckling or feeding during the immediate postnatal period. Body mass reduction in *Negr1*-I87N mutants is due to reduced lean mass. The rate of growth, as reflected in overall body weight, in homozygous male *Negr1*-I87N mutants, appears slower compared to littermates although there is some convergence later on in the time course. Despite these differences the mutants continue to gain lean mass during adulthood in a linear fashion at a rate similar to that of wild-type littermates. We observed a marked reduction in non-normalised energy expenditure in *Negr1*-I87N homozygotes, which would be expected to increase body mass counter to our observations. However, this is a secondary consequence of reduced lean mass as there is no difference in energy expenditure between genotypes when normalised to lean mass. Lean mass is relatively more metabolically active than fat mass. Physical activity in mutant mice was reduced consistent with reduced non-normalised overall energy expenditure although linear model analysis did not provide any evidence that physical activity and lean mass were directly correlated (data not shown).

*Negr1*-I87N mice display a small but significant reduction in food intake. The best fitting model for the inter-dependence of the main determinants of body mass is represented graphically in Figure S4, with food intake and genotype directly but separately related to body mass under the model. We estimated by fitting a linear model to body mass, food intake and genotype, that a total of 36% of variation in body mass across a sub-cohort of the *Negr1*-I87N cohort (comprising males and females with WT and homozygote genotypes) was explained by variation in food intake and *Negr1*-I87N genotype. A decomposition of this 36% indicated that: 7% was explained by genotype but not food intake; 22% was explained by food intake but not genotype (this is expected as any group of identical mice will vary in weight, because, for a variety of reasons, they do not each eat the same amount of food); and 7% was explained by variation shared by food intake and genotype. This latter, shared, 7% could reflect (a) genotype acting on food intake and thereby affecting body mass, (b) genotype acting on body mass and thereby affecting food intake, or (c) some other mechanism not captured by the observed variables. As expected, sex is an important determinant of body mass, mediated through food intake (males eat more). The linear modelling results indicate that the *Negr1*-I87N genotype has a relatively small effect on body mass, explaining up to 14%, compared to the 22% explained by food intake but not genotype. In our experiments, food intake is represented as ‘per day consumption’ in a ‘homecage’ environment, where animals are exposed to minimal stress. However, such acute measurements of energy expenditure or food intake are limited by the small window of the assessment time and may underestimate the true effects of genotype on food intake. These observed differences may be small but the effect of food intake on body mass is cumulative over time, which is reflected in the highly significant difference in both body mass and lean mass between the genotypes. A study in a healthy Dutch female population of the *NEGR1* SNP rs2568958 did not show statistical significant associations with weight, BMI or waist circumference, but was found to associate with dietary intake based on food frequency questionnaire. While there was no significant difference in the overall energy intake, there was a decrease in monounsaturated fat intake (−0.40 g/day, *P* = 0.03) and saturated fat intake (−0.34 g/day, *P* = 0.03) in the risk-allele carriers [10]. While this finding is consistent with our observations of an effect on food intake, in another study of similar design [47], no evidence in feeding behaviour or other lifestyle measurements were found in the risk-allele carriers.

Contrary to our expectations, we found a small but significant reduction in the daily energy loss via faeces in the NEGR1-deficient mice. Nevertheless, this finding is not necessarily contradictory to the body mass phenotype. The regulation of energy balance is highly complex and the overall metabolic state of an animal reflects the sum of the contributing components. Homozygous male mice eat less but for unknown reasons are more efficient at recovering energy during digestion. This increase in efficiency is not enough to counteract negative contributors to the balance.

While the expression of *NEGR1* has been implicated as a central ‘hub’ in an obesity-related transcription network [43], immunoblotting analysis could not reveal expression of NEGR1 in wild-type subcutaneous white adipose tissue or tissues other than the brain (Fig. S5A). This further supports the neuronal link of NEGR1 to metabolism and energy homeostasis. Nevertheless, the mechanism by which the neuronal function of NEGR1 is linked to body mass change will require further study. The available *in vitro* data on the role of NEGR1 and other IgLONs in cell-cell adhesion, neurite growth [1], [21], [22] and synapse formation [18] suggest a function of IgLONs for neuronal connectivity in the CNS, which is supported by phenotypic analysis of mice deficient for the IgLON-member LSAMP [24], [25]. The broad CNS expression of NEGR1 suggests that its function is likely to be involved in various brain regions and neuronal circuits. Nevertheless, the overall neuroanatomy of *Negr1*-KO mice showed no differences compared to wild-type littermates, as assessed by standard histological analyses (data not shown). Despite strong hypothalamic expression of NEGR1, our neuroanatomical studies in NEGR1-deficient mice showed neither a difference in the number of TH-positive neurons in the PVN or ARC nor virtual abnormalities in the projections of NPY-positive axons from the ARC to the PVN (Fig. S6A–D). Similarly, we observed no alterations in the distribution of somatostatin and corticotropin-releasing hormone (CRH)-positive axons which project to the median eminence (Fig. S6E,F). These analyses indicate that *Negr1*-KO mice lack obvious malformations in the hypothalamus. To exclude a role of NEGR1 in hypothalamic circuit formation, additional studies of neuronal subpopulations and their projections within and outside the hypothalamus are required. It also remains an open question whether NEGR1 is required for synapse formation and function *in vivo* and further analyses, including electrophysiological as well as ultrastructural studies are required in the future. Along this line, the function of NEGR1 in the brain may be best studied by mouse models with neuronal-specific or hypothalamus-specific modification of NEGR1 expression, such as by adenoviral or conditional Cre recombinase technology in combination with a floxed *Negr1* allele.

The at-risk allele of the associated variant (rs2568958) is reported to confer a 12.1% (*P*<<0.0001) per allele copy increase in *NEGR1* expression in blood [3], suggesting that up-regulation of *NEGR1* in human may have a positive effect on body mass (BMI and weight). In contrast, the loss of NEGR1 function in our mouse models appears to have a negative effect on body mass and lean mass, supporting the hypothesis that NEGR1 function may contribute to a gain in body mass. Most studies have examined weight or BMI, however, one small study has used dual-energy X-ray absorptiometry in a group of adults from northern Sweden [48]. Interestingly, this study found that the variance in weight associated with the *NEGR1* rs2815752 was determined to a larger extent by non-adipose tissue (i.e. lean mass) in addition to a lesser contribution by adipose mass. This is consistent with the data from our *Negr1*-I87N mice where lean mass is also the major determinant.

In summary, our novel mouse models provide evidence to support a role for *NEGR1* in the control of body weight and composition at least partly through alterations in food intake and add to the support for *NEGR1* as the gene underlying the GWAS signal in the human studies.

## Materials and Methods

### Ethics statement

*Negr1*-KO mice were kept according to the principles of good laboratory animal care and in approval by local authorities (Regierungspräsidium Freiburg). *Negr1*-I87N mice were kept in accordance with UK Home Office welfare guidelines, project license restrictions and approval by local Ethics Committee.

### Antibodies and cDNA constructs

The following antibodies were used for immunoblot (IB), immunohistochemistry (IHC) or immunocytochemistry (ICC): mouse anti-β-actin (Sigma, IB, diluted 1∶10.000), rabbit anti-calnexin (Abcam, IB, diluted 1∶1000), rabbit anti-CRH (UCB Bioproducts, IHC, diluted 1∶200), mouse anti-GFP (Millipore, ICC, diluted 1∶1000), rabbit anti-NEGR1 (Sigma, IB, diluted 1∶2000; ICC, diluted 1∶300), rabbit anti-NPY (Immunostar, diluted 1∶2000); rabbit anti-somatostatin (Chemicon, IHC, diluted 1∶1500), rabbit anti-tyrosine hydroxylase (IHC, diluted 1∶2000), mouse anti-βIII tubulin (Covance, ICC, diluted 1∶1000), Cy3- and Alexa 488 Fluor- conjugated secondary antibodies (Molecular Probes, IHC, ICC 1∶500) and HRP-conjugated secondary antibody (Jackson ImmunoResearch, IB 1∶5000). cDNA constructs used in this study: DsRed-ER, pEGFP-C1 (Clontech). To obtain pcDNA3-NEGR1 the cDNA was subcloned from pBSKS-Neurotractin/NEGR1 [22]. The pcDNA3-*Negr1*-I87N plasmid was generated from the pcDNA3-NEGR1 plasmid using QuikChange Lightning Site-Directed Mutagenesis kit (Stratagene) with the following primer containing the I87N mutation, and confirmed by DNA sequencing:

*Negr1*-I87N-F 5′-CAGTGGACCCTCGAGTTTCCATTTCCACATTGAATAAAAGAG-3′

*Negr1*-I87N-R 5′-GTCTCTTTTATTCAATGTGGAATTGGAAACTCGAGGGTCCAC-3′

### Immunohistochemistry and *in situ* hybridisation

For immunohistochemistry, mice were anaesthetised and transcardially perfused with 4% paraformaldehyde. Dissected brains were postfixed overnight and cryoprotected in 30% sucrose for 20 hours at 4°C and processed for sectioning as described [49]. Images were captured using an inverted light microscope (Olympus BX60), equipped with a monochrome digital camera (Leica, DFC350FX). *In situ* hybridisation was performed essentially as described [50]. Sense and anti-sense probes were generated against full length murine *Negr1* cDNA, sequences are available on request.

### Stereological cell counts

Stereological cell counts (Stereo Investigator software: MicroBrightField, Inc., Williston, VT; version 4.31) were performed for TH-immunoreactive neurons in the hypothalamic PVN and ARC. The optical dissector/fractionator method was applied as described in detail previously [51].

### SDS-Page, immunoblotting, cell culture and immunocytochemistry

SDS-Page and immunoblot analyses as well as the culture of NSC-34 cells and preparation of primary neurons were essentially performed as described [34]. Cells were transfected with cDNA constructs using polyethylenimine [52]. Transfection efficiency was about 30–35% in all experiments (data not shown). To determine subcellular localization of wild-type and mutant NEGR1, high magnification images from transfected NSC-34 cells were acquired using a LSM510 confocal laser scanning microscope with a 63× objective and appropriate filters (Zeiss). For co-culture experiments, NSC-34 cells were grown on poly-D-lysine coated glass coverslips in 24-well plates to about 70% confluency and co-transfected with pEGFP-C1, together with pcDNA3-*Negr1*-WT or pcDNA3-*Negr1*-I87N. Cells expressing pEGFP alone served as control. After 16 h, primary hypothalamic neurons were prepared from newborn mice and 50.000 neurons were seeded onto confluent monolayers of NSC-34 cells and co-cultured for 48 hrs. Then, cultures were fixed in 4% PFA for 30 min, blocked in 5% goat serum/0.5% BSA/0.1% TX-100 and immunostained using antibodies specific to βIII-tubulin. To quantify neurite lengths in co-cultures of NSC-34 cells and primary neurons, identical numbers of images were captured for each experiment and condition using an inverted light microscope (Olympus BX60), equipped with a monochrome digital camera (Leica, DFC350FX) and a 10×objective (Olympus). Images were then processed for neurite length measurements using ImageJ.

### Lasercapture microdissection and quantitative PCR

Coronal brain sections of 15 µm were cut in a cryostat and collected on polyethylene terephthalate membrane slides (Leica). Sections were dried at 30°C for 45 min, stepwise dehydrated in 50%, 75%, 95%, and 100% ethanol (10 sec each) and stained by 0.1% cresyl violet dissolved in 100% ethanol for 2 min. Hypothalamic nuclei were dissected from serial sections using a Leica laser microdissection microscope. Microdissected nuclei were collected in Trizol reagent (Invitrogen) and RNA isolated by Phenol:Chloroform extraction. cDNA for qPCR was prepared using a Verso RT-PCR Kit (Abgene). qPCR was performed on a BioRad light cycler using SYBR Green Mastermix (Abgene). Gene expression levels were normalized to the expression of the housekeeping gene*Polr2A*and analyzed by the comparative ΔC_T_ method. Primer for *Polr2A* were purchased from Qiagen (QuantiTect Primer Assay). Primer sequences used for the amplification were as follows:

5′-ATGTGACGCAGGAGCACTT-3′(*forward Negr1*);

5′-CCATACTGGGCTGTACTTGGA-3′(*reverse Negr1*);

5′-GGCAAGAGATCCAGCCCTG-3′(*forward NPY*);

5′-CCAGCCTAGTGGTGGCATGC-3′(*reverse NPY*);

### Cell aggregation assay

NSC-34 cells were grown and transfected with pcDNA3-*Negr1*-WT or pcDNA3-*Negr1*-I87N together with pEGFP-C1 (Clontech). Co-expression of pEGFP was observed in at least 80–90% of NEGR1-positive cells in all experiments and conditions (data not shown). Cell aggregation assay was essentially performed as described [53] and quantified by determining the increase of average areas of green fluorescent cells and aggregates between 0 min (t1) and 60 min (t2) in each condition using Image J software. The calculated t2/t1 ratios were expressed relative to control cells expressing pEGFP alone.

### Generation of *Negr1* mouse alleles (ENU and KO)

To disrupt the *Negr1* gene, we generated a targeting vector that replaced exon 2 including the 3′splice site with a neomycin cassette. To generate the targeting vector, a murine genomic BAC library derived from from a mouse 129/SvJ II ES cell line clones was screened by PCR using degenerated primers (5′-GTG ACA AGT GGT CRG TGG ACC C-3′ and GTG TGY TGG GTY TGC ACA GAA, R = A/G; Y = T/C) for the presence of exon 2 of the murine *Negr1* gene (in collaboration with Incyte Genomics). Positively screened BAC clones were mapped by restriction enzyme digestion and two appropriate fragments of 890 bp (short arm) and 8.3 kb (long arm) subcloned into the pTV0 vector (kindly provided by Carmen Birchmeier, Max-Delbrück-Center for Molecular Medicine, Berlin, Germany). Then, the *Negr1*-KO allele was generated by homologous recombination in E14.1 embryonic stem cells [54]. Electroporation, selection and blastocyst injection of E14.1 ES cells were performed according to standard protocols. Three targeted ES cell clones were identified by genomic PCR and transmission of the targeted *Negr1* locus confirmed by southern blotting. Chimeric mice were bred to C57BL/6 mice and progeny were identified by coat color. Heterozygous *Negr1* founders were back-crossed into the C57BL/6J strain for 8 generations. Mice were genotyped by genomic PCR using the following primers: forward 5′CAC TGC AGA AGG CAA CAA TC 3′; reverse 5′CCT TCT CTA GCC ATG CTT TGT AC 3′ resulting in 2.7 kb (wild-type allele) and 2.8 kb (mutant allele) amplification products.

The Harwell ENU-DNA archive was screened for mutations in *Negr1* by high resolution melting (HRM) DNA analysis method using the LightScanner system (Idaho Technology, Salt Lake City, UT, USA). *Negr1*-I87N is one of the ENU mutations identified in PCR products amplified from the DNA samples of ∼10,000 G1 mice with the following primers: *Negr1*ex2F 5′TCC TTC CCT TCC TCC ATA CC-3′ and *Negr1*ex2R 5′CTC AGT ATT TCA TTT CAA GCT TAT CC-3′. *Negr1*-I87N animals were subsequently generated using frozen sperm samples from BALB/c×C3H/HeH F1 founder and C3H/HeH eggs through *in vitro* fertilisation [32]. Progeny were backcrossed for six generations to C3H/HeH and intercrossed to produce mice heterozygous and homozygous for the mutation. NEGR1 peptide sequences were exported from Ensembl (www.ensembl.org). Multiple sequence alignment was performed with ClustalW.

### Animal Husbandry

Animals were kept under controlled light (12 hr light and 12 hr dark cycle, dark 7pm–7am), temperature (21°C±2°C) and humidity (55%±10%) conditions. They had free access to water (25 ppm chlorine) and were fed *ad libitum* on a commercial high-fat diet (containing 45 kcal% fat, 20 kcal% protein and 35 kcal% carbohydrate - D12451, Research Diets, New Brunswick, NJ, USA) or a standard diet (containing 11.5 kcal% fat, 23.93 kcal% protein and 61.57 kcal% carbohydrate - SDS Rat and Mouse No. 3 Breeding diet). All mice for the *Negr1*-I87N experiments were supplied by MRC Harwell, Harwell Oxford, England. Phenotypic analyses were performed in accordance with the standardized operating protocols in EMPReSS (European Phenotyping Resource for Standardized Screens from EUMORPHIA, http://empress.har.mrc.ac.uk).

### Metabolic phenotyping of *Negr1*-I87N mice

Body mass was measured weekly on an electronic scale calibrated to 0.01 g. Fat mass and lean mass were obtained fortnightly in conscious mice by quantitative magnetic resonance imaging (EchoMRI, Echo Medical Systems, Houston Texas, USA) with calibration to canola oil for fat mass measurement. Metabolic rate was measured at 12 weeks of age using indirect calorimetry (Oxymax; Columbus Instruments) to determine oxygen consumption (VO_2_), carbon dioxide production (VCO_2_), respiratory exchange ratio (RER) and heat production. Food and water intake, metabolic rate and physical activity were further measured at 16 wk in a “home-cage-like” Phenomaster system (TSE Systems, Bad Homburg, Germany), which consists of feeding and drinking sensors for automated measurement, indirect calorimetry measurements, and a photobeam-based activity monitoring system that detects all movements. All parameters were measured continuously and simultaneously for 24 hours in singly housed mice. At 22 weeks of age, mice fasted for ∼8 h were killed by anesthetic overdose, and blood collected by cardiac puncture. Plasma concentrations of triglycerides, glycerol, free fatty acids, total cholesterol, HDL cholesterol, ketone bodies, lactate dehydrogenase, creatine kinase, ALP, ALT and AST were measured on an AU400 (Olympus UK), as described [55]. Faeces samples of individually caged mice were collected over 2 days. Samples of up to 5 mice of the same sex and genotype were pooled for bomb calorimetry (IKA7000, Ika Staufen, Germany) resulting in 5–6 pooled samples for combustion per group.

### Histology

The median lobe of liver were dissected and fixed in 10% neutral buffered formalin. Paraffin-embedded sections of 3 microns were stained with hematoxylin and eosin. Photomicrographs were captured by optic microscopy (Zeiss Axiostar Plus) with the ALTRA20 Soft Imaging System (Olympus). The diameter of triglyceride droplets in hepatocytes were measured for two fields per animal at ×10 and ×40 magnification respectively, with the cell∧B imaging software (Soft Imaging System, Olympus).

### Time-course data analysis

Time-course data were analysed using two complementary methods [36], (i) repeated measures ANOVA, and (ii) time-by-time ANOVA.

#### Repeated measures ANOVA

The following repeated-measures ANOVA model [36] was fitted to each phenotype's data across all three genotypes and across all time points.


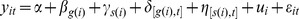


in which

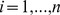
 indexes mouse,

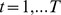
 indexes time point,

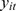
 denotes the phenotype of mouse 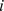 at time point 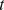,

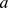
 denotes the intercept term

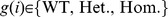
 denotes the genotype of mouse 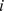,

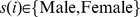
 denotes the sex of mouse 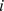,
- the 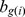 denote the main genotypic effects (with the constraint 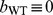)
- the 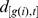 denote interactions between genotype and time (with the constraints 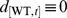 and 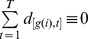),
- the 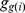 denote the main sex effect (with the constraint 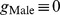),
- the 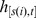 denote interactions between sex and time (with the constraints 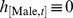 and 
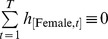
),

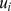
 is the random effect for mouse 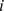 (these random effects model the correlation between repeated measures), with the 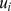 assumed to be mutually independent identically distributed zero-mean Gaussian random variables, and
- the 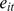 are mutually independent identically distributed zero-mean Gaussian random measurement errors.

The model was fitted using the lmer() function in the lme4 R package [56], [57]. Estimates (with standard errors) for the 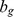, are shown in Tables 1 and 2. The null hypothesis of no main genotypic effect (i.e. that 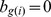 for all genotypes) was tested against the alternative hypothesis under which 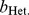 and 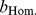 were unconstrained. The test was based on the asymptotic 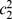 null distribution of 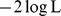, where 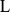 denotes the likelihood ratio.

The model above was applied to data from both genders combined, and thereby had relatively high statistical precision and power (compared to a sex-specific model) when the assumptions of the model were justified, and in particular when the genotypic effects were the same in both genders. Additionally, in order to characterize sex-specific genotype effects, the following model was fitted to each gender separately:


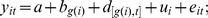


The results of the sex-specific analysis are shown in Tables 1 and 2.

#### Time-by-time ANOVA

For each measurement time point of each phenotype, the following (two-way ANOVA) linear model was fitted:


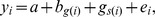


where

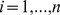
 indexes mouse,

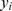
 denotes the phenotype of mouse 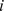,

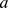
 denotes the intercept term

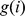
 denotes the genotype of mouse 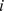,

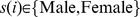
 denotes the gender of mouse 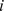,
- the 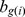 denote the genotypic effect (with the constraint 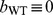)
- the 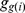 denote the gender effect (with the constraint 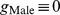),
- the 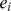 are mutually independent identically distributed zero-mean Gaussian residual error terms.

Parameter estimates were obtained, and, using the *F*-test for the ordinary linear model, the null hypothesis of no genotypic effect (i.e. 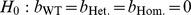) was tested against the alternative hypothesis under which 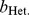 and 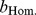 were unconstrained. Parameter estimates, standard errors, and p-values are shown in Table S1.

The model above was fitted to data from both genders collectively, thereby increasing the precision to estimate, and power to detect, genotypic effects when that model holds, and in particular when the genotypic effects are the same in both genders. In order to go on to investigate genotypic effects that differed between genders, the following sex-specific one-way ANOVA model was fitted to each gender's data separately:


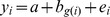


To account for multiple testing across time points and across the three model fits (males, females, and both genders combined), a Bonferroni correction was applied to the p-values resulting from each phenotype's analysis, correcting for a total of 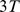 tests, where 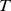 denotes the number of time points; nominal and corrected p-values are shown in Table S1.

### Dependence of body mass on genotype and food intake

A mixed graphical model [40], [41] was fitted to data comprising six variables measured on 85 mice 16 weeks of age in the *Negr1*-I87N study, using the R package gRapHD. Four of the variables were quantitative (body mass, food intake, energy expenditure, and physical activity), and two were qualitative (sex and genotype, with only homozygotic and wild-type genotypes represented in the sample used for this part of the study. Prior to model fitting, the physical activity (number of beambreaks in the horizontal plane, XT+YT) was log-transformed to make its distribution more Gaussian. The default fitting method in the gRapHD package was used (it was based on the Bayesian information criterion, or BIC). The fitted graph is shown in Figure S4.

A linear model was fitted, with body mass as the response variable, and food intake and genotype as the explanatory variables, i.e.


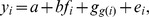


where 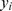, 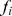, and 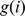 denote the body mass, food intake, and genotype (respectively) of mouse 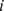. The food intake and genotype effects are parameterized by 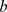 and 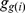 respectively, 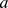 is an intercept term, and the 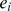 are independent, identically distributed, Gaussian residual error terms. Parameter estimates, standard errors, *t*-statistics, and *t*-test p-values are shown in Table S2; multiple 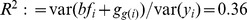, i.e. 36% of variation in body mass was explained by the two explanatory variables. The numerator was decomposed to provide a decomposition of 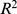 into three components attributable to: (i) food intake alone (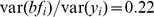); (ii) genotype alone (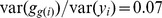); and (iii) variation shared by food intake and genotype (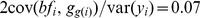).

## Supporting Information

Figure S1 **NEGR1-I87N is highly conserved across vertebrate species.** **A**. Schematic structure of NEGR1 and the location of the I87N mutation as indicated (C2, Ig-like C2-type domain). **B**. Multiple sequence alignment showing that NEGR1-I87N (blue arrow) is conserved across a wide range of species. Residues conserved to mouse NEGR1 are shaded in yellow.
(TIF)

Figure S2 **Association of body mass and lean mass with energy expenditure in** ***Negr1*** **-I87N mice.** **A–B, E–F**. Association of body mass with energy expenditure during light phase (A,E) and dark phase (B,F) in female (A–B) and male (E–F) mice. **C–D, G–H**. Association of lean mass with energy expenditure during light phase (C,G) and dark phase (D,H) in female (C–D) and male (G–H) mice. The lines are the best fit of a straight line through the data using linear regression analysis. p-values for differences in the slope (S) and the elevation or intercept (E/I) of the lines are against wild-type mice (GraphPad Prism).
(TIF)

Figure S3 **Analysis on faecal mass and energy content in** ***Negr1*** **-I87N mice.** **A–C**. Faecal content represented in grams (A), normalized to body mass (B), and as energy content per gram (C) in female (WT, n = 27; het, n = 22; hom, n = 26) and male (WT, n = 22; het, n = 22; hom, n = 24) *Negr1*-I87N mice at 14 weeks of age. Data are presented as mean ± SEM.
(TIF)

Figure S4 **Mixed graphical model for data collected at time point 16 weeks of** ***Negr1*** **-I87N study.** Conditional dependence between variables (shown as vertices in the graph) is represented by the edges in the graph, so, for example, “X and Y are conditionally independent given Z,” is represented by the graphical property “all paths joining X and Y pass through Z.” See Methods and [58], [59] for further details.
(TIFF)

Figure S5 **Hypothalamic** ***Negr1*** **expression is unaffected by fasting.** **A.** Absence of NEGR1 expression in peripheral tissues. Blots were probed with antibodies specific to NEGR1 (∼50 kDa, upper panel) and α-actin (43 kDa, lower panel). The protein band in muscle represents a ∼45 kDa non-NEGR1-specific soluble protein that is resistant to deglycosylation by PNGaseF (data not shown). **B, C.** Nissl-stained sections of mouse hypothalamus before and after LCM of PVN (B) and ARC (C) tissue. **D, E.** Quantitative PCR from LCM samples from PVN (ctl, n = 9; fast, n = 5) and ARC (ctl, n = 8; fast, n = 8) for *Negr1* (D) and *Npy* (E) normalized to *PolR2A*. *p = 0.041, Student's *t*-test. No statistically significant differences for normalized *Negr1* expression were obtained between different hypothalamic nuclei and feeding conditions (control = ctl; 24 hr fasting = fast). All data are presented as mean ± SEM. co, cerebral cortex; hy, hypothalamus; oes, oesophagus; sto, stomach; duo, duodenum; ile, ileum; col, colon; cae, caecum; rec, rectum; WAT, white adipose tissue; ins, intestinal WAT; sub, subsutaenous WAT; epi, epigonadal WAT; pan, pancreas; spl, spleen; liv, liver; kid, kidney; mus, muscle; BAT, brown adipose tissue.
(TIF)

Figure S6 **Normal brain anatomy in** ***Negr1*** **-KO mice.** **A, B.** Distribution of NPY-positive axons in the PVN and ARC. **C.** Immunostaining for TH-positive neurons in the PVN and ARC. **D.** Graph showing relative number of TH-positive neurons in PVN and ARC of wild-type and KO mice (n = 4/genotype). Data are presented as mean ± SEM. Student's *t*-test (2-tailed) was carried out between groups. **E.** Immunostaining for somatostatin-positive axons in the ventromedial nucleus (VMN), ARC and median eminence. **F.** CRH-immunostaining in the median eminence.
(TIF)

Table S1 **Time-by-time ANOVA results describing phenotypic dependence on sex and genotype.**
(XLS)

Table S2 **Details of a fitted linear model, in which body mass was the response variable, and food intake and genotype were the explanatory variables.**
(XLS)
