# Supplementary figures and images for: Functional Inactivation of the Genome-Wide Association Study Obesity Gene Neuronal Growth Regulator 1 in Mice Causes a Body Mass Phenotype
Source: PLoS One. 2012 Jul 23;7(7):e41537. doi: 10.1371/journal.pone.0041537 (PMC3402391; doi:10.1371/journal.pone.0041537)

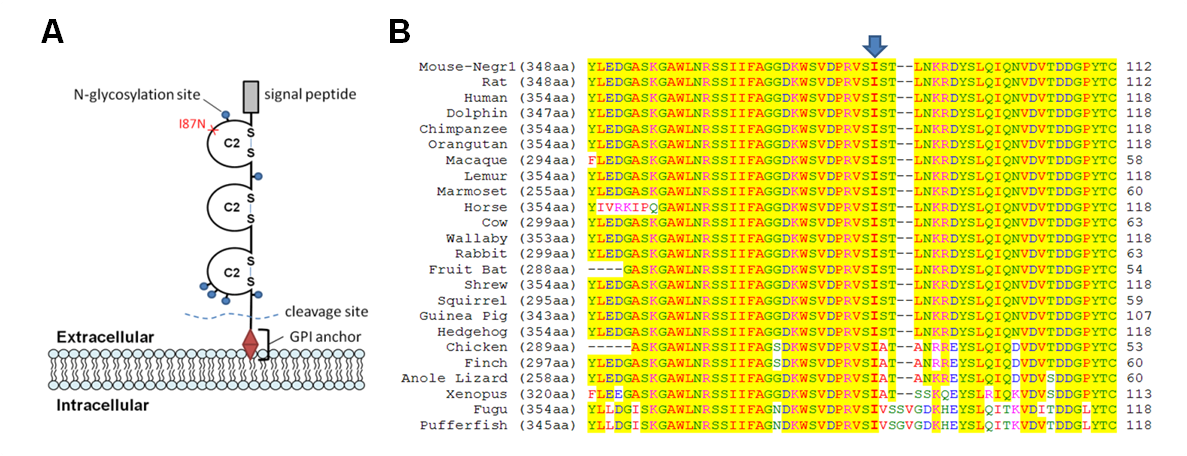

Supplement: Figure S1 — NEGR1-I87N is highly conserved across vertebrate species. A. Schematic structure of NEGR1 and the location of the I87N mutation as indicated (C2, Ig-like C2-type domain). B. Multiple sequence alignment showing that NEGR1-I87N (blue arrow) is conserved across a wide range of species. Residues conserved to mouse NEGR1 are shaded in yellow. (TIF) [file pone.0041537.s001.tif]

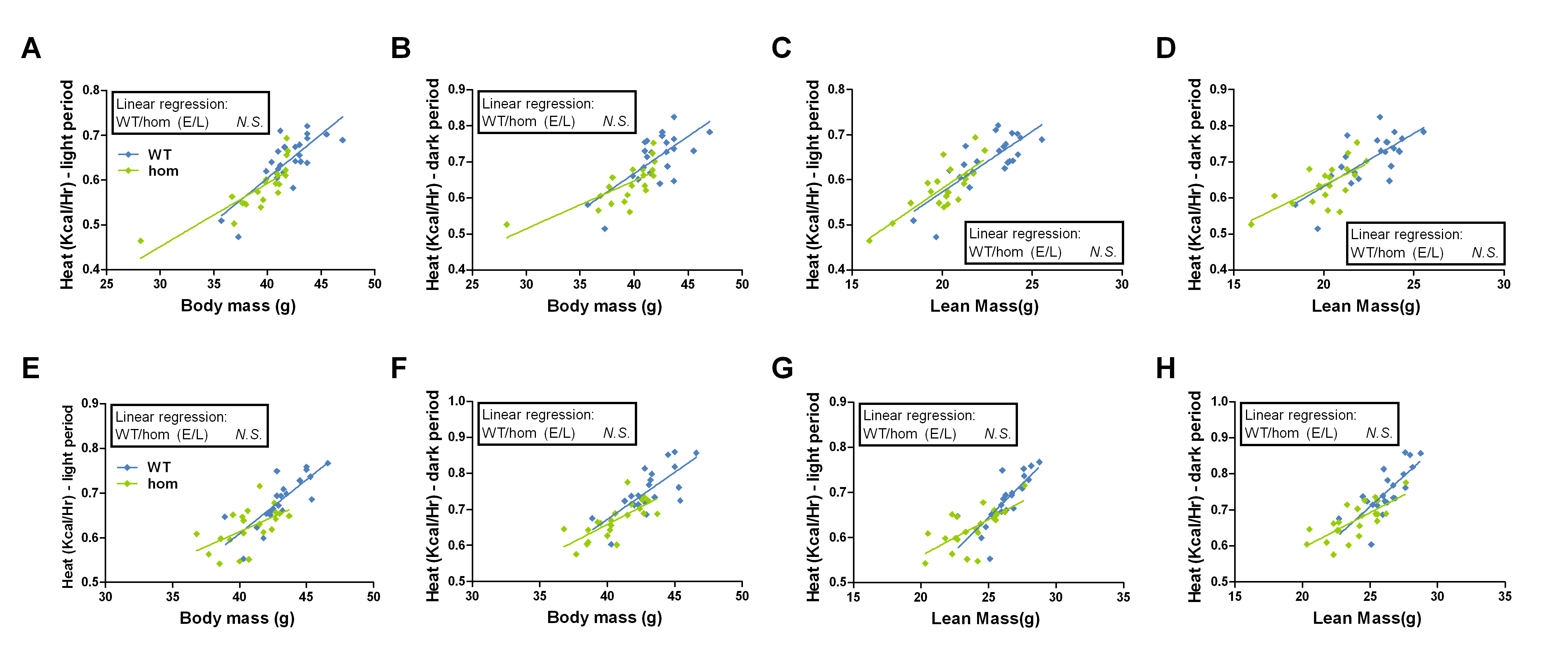

Supplement: Figure S2 — Association of body mass and lean mass with energy expenditure in Negr1 -I87N mice. A–B, E–F. Association of body mass with energy expenditure during light phase (A,E) and dark phase (B,F) in female (A–B) and male (E–F) mice. C–D, G–H. Association of lean mass with energy expenditure during light phase (C,G) and dark phase (D,H) in female (C–D) and male (G–H) mice. The lines are the best fit of a straight line through the data using linear regression analysis. p-values for differences in the slope (S) and the elevation or intercept (E/I) of the lines are against wild-type mice (GraphPad Prism). (TIF) [file pone.0041537.s002.tif]

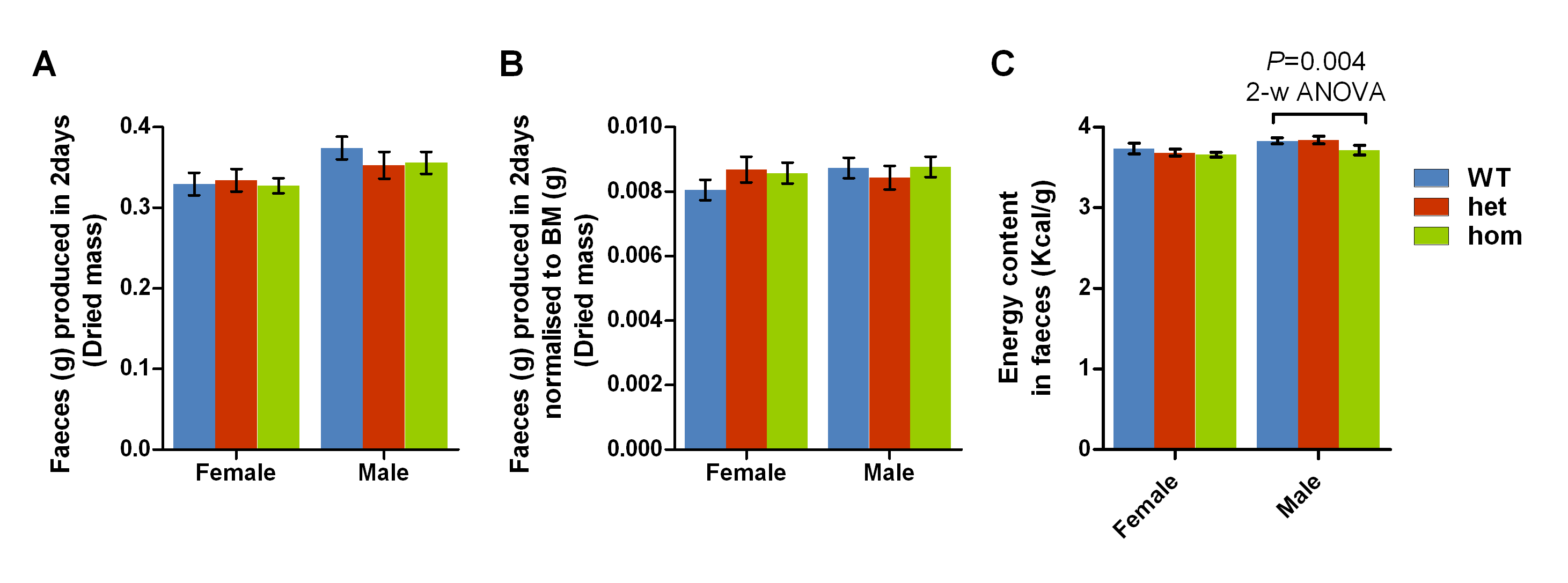

Supplement: Figure S3 — Analysis on faecal mass and energy content in Negr1 -I87N mice. A–C. Faecal content represented in grams (A), normalized to body mass (B), and as energy content per gram (C) in female (WT, n = 27; het, n = 22; hom, n = 26) and male (WT, n = 22; het, n = 22; hom, n = 24) Negr1-I87N mice at 14 weeks of age. Data are presented as mean ± SEM. (TIF) [file pone.0041537.s003.tif]

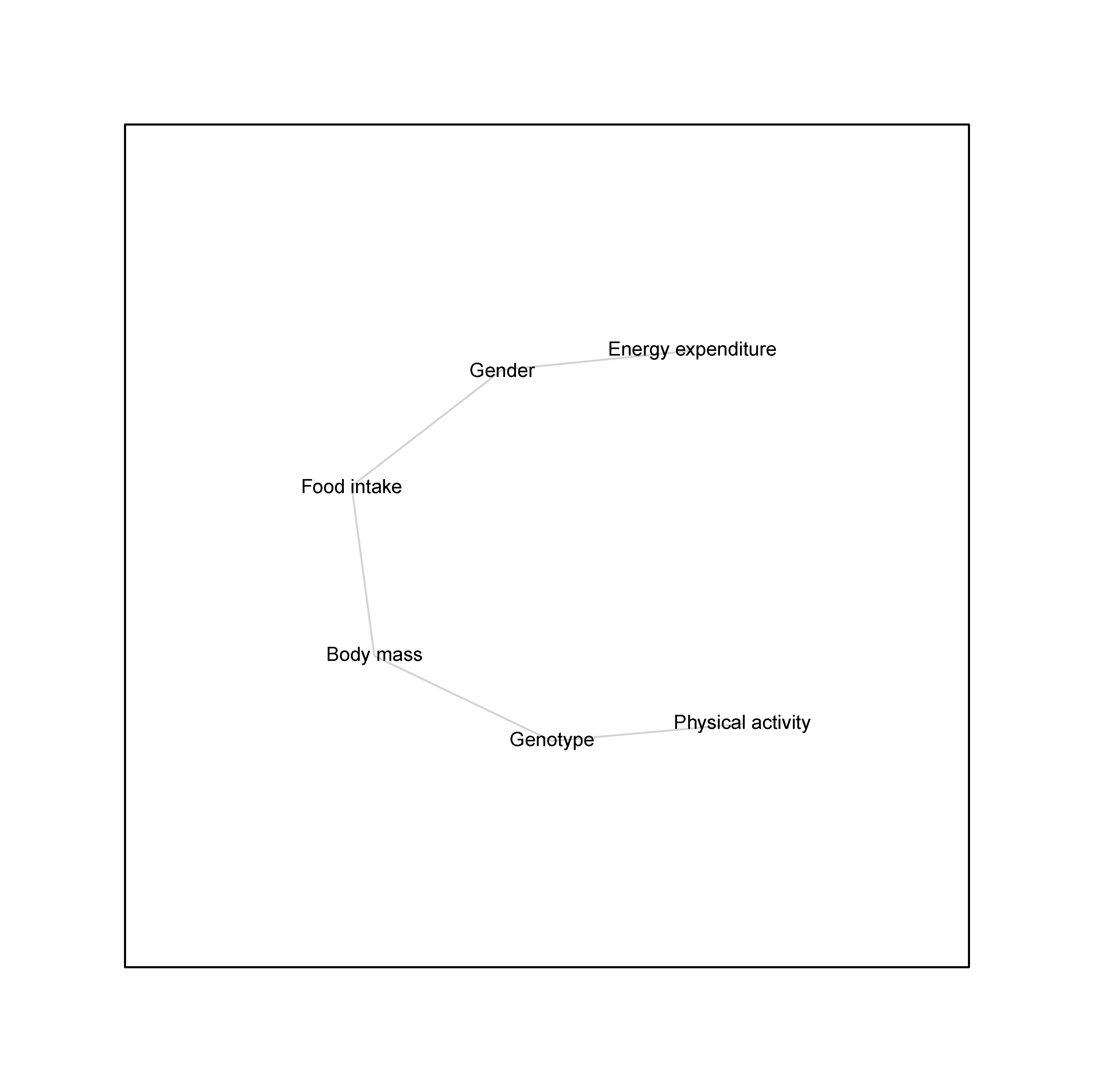

Supplement: Figure S4 — Mixed graphical model for data collected at time point 16 weeks of Negr1 -I87N study. Conditional dependence between variables (shown as vertices in the graph) is represented by the edges in the graph, so, for example, “X and Y are conditionally independent given Z,” is represented by the graphical property “all paths joining X and Y pass through Z.” See Methods and [58], [59] for further details. (TIFF) [file pone.0041537.s004.tiff]

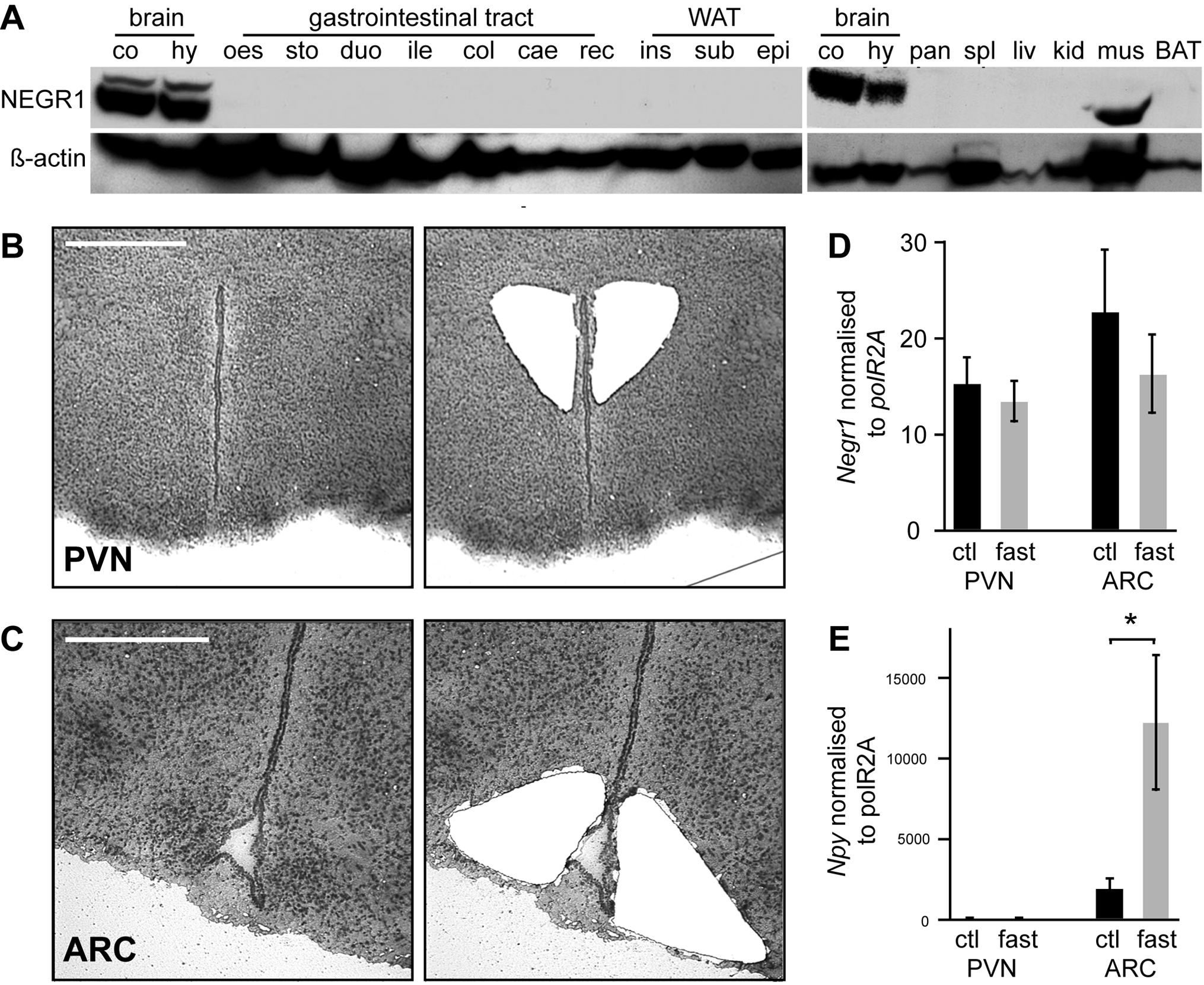

Supplement: Figure S5 — Hypothalamic Negr1 expression is unaffected by fasting. A. Absence of NEGR1 expression in peripheral tissues. Blots were probed with antibodies specific to NEGR1 (∼50 kDa, upper panel) and α-actin (43 kDa, lower panel). The protein band in muscle represents a ∼45 kDa non-NEGR1-specific soluble protein that is resistant to deglycosylation by PNGaseF (data not shown). B, C. Nissl-stained sections of mouse hypothalamus before and after LCM of PVN (B) and ARC (C) tissue. D, E. Quantitative PCR from LCM samples from PVN (ctl, n = 9; fast, n = 5) and ARC (ctl, n = 8; fast, n = 8) for Negr1 (D) and Npy (E) normalized to PolR2A. *p = 0.041, Student's t-test. No statistically significant differences for normalized Negr1 expression were obtained between different hypothalamic nuclei and feeding conditions (control = ctl; 24 hr fasting = fast). All data are presented as mean ± SEM. co, cerebral cortex; hy, hypothalamus; oes, oesophagus; sto, stomach; duo, duodenum; ile, ileum; col, colon; cae, caecum; rec, rectum; WAT, white adipose tissue; ins, intestinal WAT; sub, subsutaenous WAT; epi, epigonadal WAT; pan, pancreas; spl, spleen; liv, liver; kid, kidney; mus, muscle; BAT, brown adipose tissue. (TIF) [file pone.0041537.s005.tif]

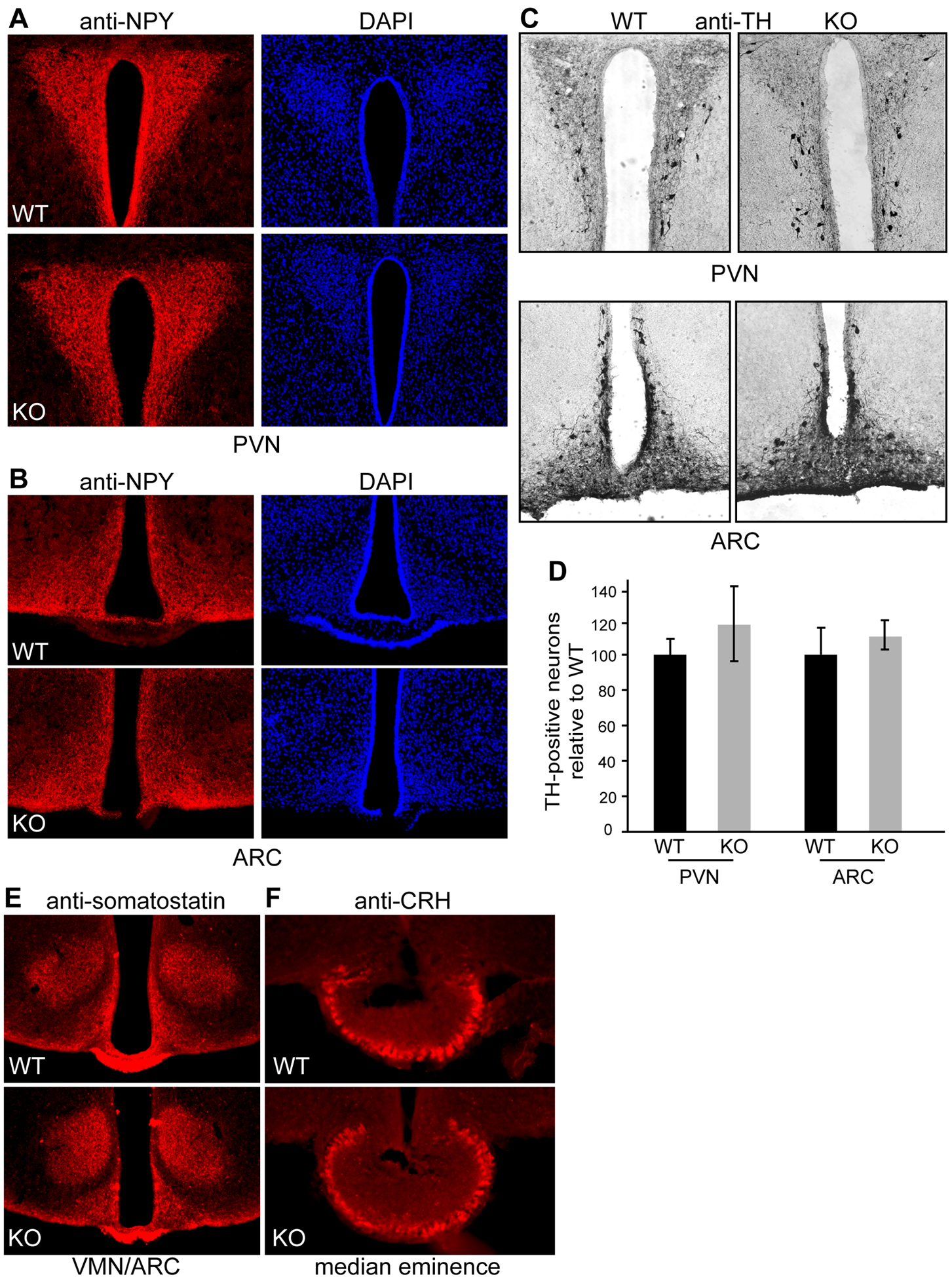

Supplement: Figure S6 — Normal brain anatomy in Negr1 -KO mice. A, B. Distribution of NPY-positive axons in the PVN and ARC. C. Immunostaining for TH-positive neurons in the PVN and ARC. D. Graph showing relative number of TH-positive neurons in PVN and ARC of wild-type and KO mice (n = 4/genotype). Data are presented as mean ± SEM. Student's t-test (2-tailed) was carried out between groups. E. Immunostaining for somatostatin-positive axons in the ventromedial nucleus (VMN), ARC and median eminence. F. CRH-immunostaining in the median eminence. (TIF) [file pone.0041537.s006.tif]
